# Management of Coronary Artery Disease in Older Adults: Recent Advances and Gaps in Evidence

**DOI:** 10.3390/jcm12165233

**Published:** 2023-08-11

**Authors:** Stefano Cacciatore, Luigi Spadafora, Marco Bernardi, Mattia Galli, Matteo Betti, Francesco Perone, Giulia Nicolaio, Emanuele Marzetti, Anna Maria Martone, Francesco Landi, Elad Asher, Maciej Banach, Olivier Hanon, Giuseppe Biondi-Zoccai, Pierre Sabouret

**Affiliations:** 1 Department of Geriatrics, Orthopedics and Rheumatology, Università Cattolica del Sacro Cuore, Largo F. Vito 1, 00168 Rome, Italy; 2 Department of Clinical, Internal Medicine, Anesthesiology and Cardiovascular Sciences, Sapienza University of Rome, Viale del Policlinico 155, 00186 Rome, Italy; 3 Maria Cecilia Hospital, GVM Care & Research, 48033 Cotignola, Italy; 4 University of Milan, 20122, Milan, Italy; 5 Monzino IRCCS Cardiological Center, 20137 Milan, Italy; 6 Cardiac Rehabilitation Unit, Rehabilitation Clinic “Villa delle Magnolie”, 81020 Castel Morrone, Caserta, Italy; 7 Department of Experimental and Clinical Medicine and Geriatrics, University of Florence, Azienda Ospedaliero Universitaria Careggi, Largo Giovanni Alessandro Brambilla 3, 50134 Florence, Italy; 8 Fondazione Policlinico Universitario “Agostino Gemelli” IRCCS, Largo A. Gemelli 8, 00168 Rome, Italy; 9 The Jesselson Integrated Heart Center, Shaare Zedek Medical Center, Faculty of Medicine, Hebrew University, P.O. Box 12271, Jerusalem 9112102, Israel; 10 Department of Preventive Cardiology, Polish Mother’s Memorial Hospital Research Institute (PMMHRI), Medical University of Lodz (MUL), 93-338 Lodz, Poland; 11 Assistance Publique Hôpitaux de Paris, Geriatric Department, Broca Hospital, University of Paris Cité, 54–56 Rue Pascal, 75013 Paris, France; 12 Department of Medical-Surgical Sciences and Biotechnologies, Sapienza University of Rome, Corso della Repubblica 79, 04100 Latina, Italy; 13 Mediterranea Cardiocentro, Via Orazio 2, 80122 Naples, Italy; 14 Heart Institute, Pitié-Salpétrière Hospital, ACTION-Group, Sorbonne University, 47–83 Bd de l’Hôpital, 75013 Paris, France; 15 Department of Cardiology, National College of French Cardiologists, 13 Rue Niépce, 75014 Paris, France

**Keywords:** aged, frailty, coronary artery disease, ischemia, hemorrhage, multimorbidity, antithrombotic agents

## Abstract

Coronary artery disease (CAD) is highly prevalent in older adults, yet its management remains challenging. Treatment choices are made complex by the frailty burden of older patients, a high prevalence of comorbidities and body composition abnormalities (e.g., sarcopenia), the complexity of coronary anatomy, and the frequent presence of multivessel disease, as well as the coexistence of major ischemic and bleeding risk factors. Recent randomized clinical trials and epidemiological studies have provided new data on optimal management of complex patients with CAD. However, frail older adults are still underrepresented in the literature. This narrative review aims to highlight the importance of assessing frailty as an aid to guide therapeutic decision-making and tailor CAD management to the specific needs of older adults, taking into account age-related pharmacokinetic and pharmacodynamic changes, polypharmacy, and potential drug interactions. We also discuss gaps in the evidence and offer perspectives on how best in the future to optimize the global strategy of CAD management in older adults.

## 1. Introduction

**Figure 1:**
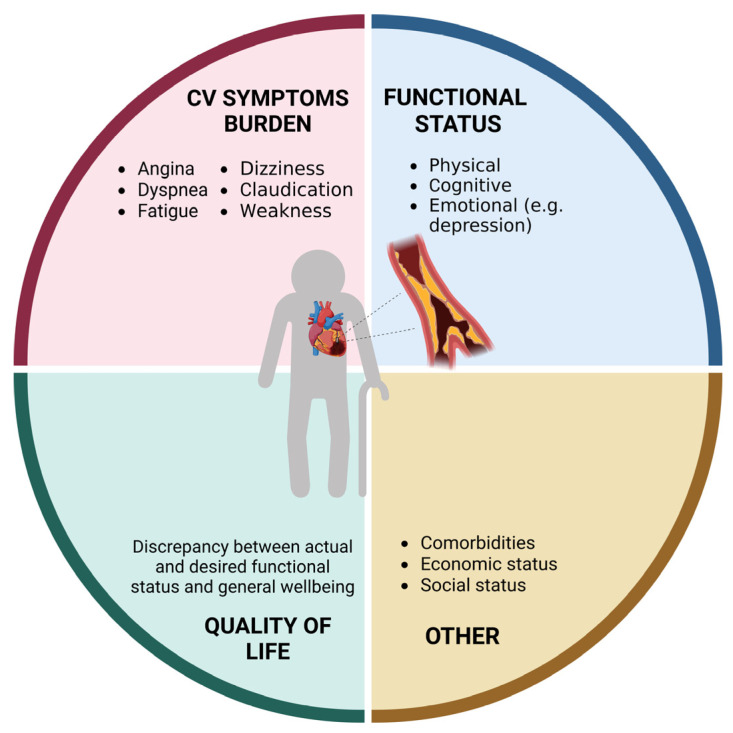
Domains affected by coronary heart disease in older adults (created with Biorender.com, accessed on 30 June 2023).

Coronary heart disease (CAD) is the leading cause of mortality worldwide [1]. Due to evolution in medical sciences and technology, life expectancy has increased over the last century. This has caused a “boomerang effect” in terms of prevalence of cardiovascular disease (CVD), with more than 60% of all cardiovascular deaths occurring in people aged 75 years and older [1]. Acute coronary syndromes (ACSs) impose a significant health burden (Figure 1) and are the most frequent cause of death in older adults. Aging, per se, increases CVD risk via several pathophysiological mechanisms, such as increased arterial and ventricular stiffness, altered blood pressure control, increased oxidative stress and inflammation levels, hypercholesterolemia, and impaired glucose metabolism [2]. Notwithstanding, older adults are still underrepresented in clinical trials testing therapeutics for CVD [3], and conventional endpoints may not be adequate for addressing the medical needs and expectations of older individuals [4]. The lack of robust evidence and the frequent presence of multimorbidity, polypharmacy, frailty, body composition abnormalities, and geriatric syndromes make the management of older patients with CVD highly complex. In this review, we highlight the main issues related to the management of CAD in older adults. We highlight the need for frailty assessment in these patients and discuss possible strategies for managing ACSs through invasive or non-invasive treatments and optimizing medical treatment. We finally discuss issues related to the follow-up of older adults with CAD, as well as gaps in the evidence and perspectives on the future.

## 2. Assessment of Frailty

**Figure 2:**
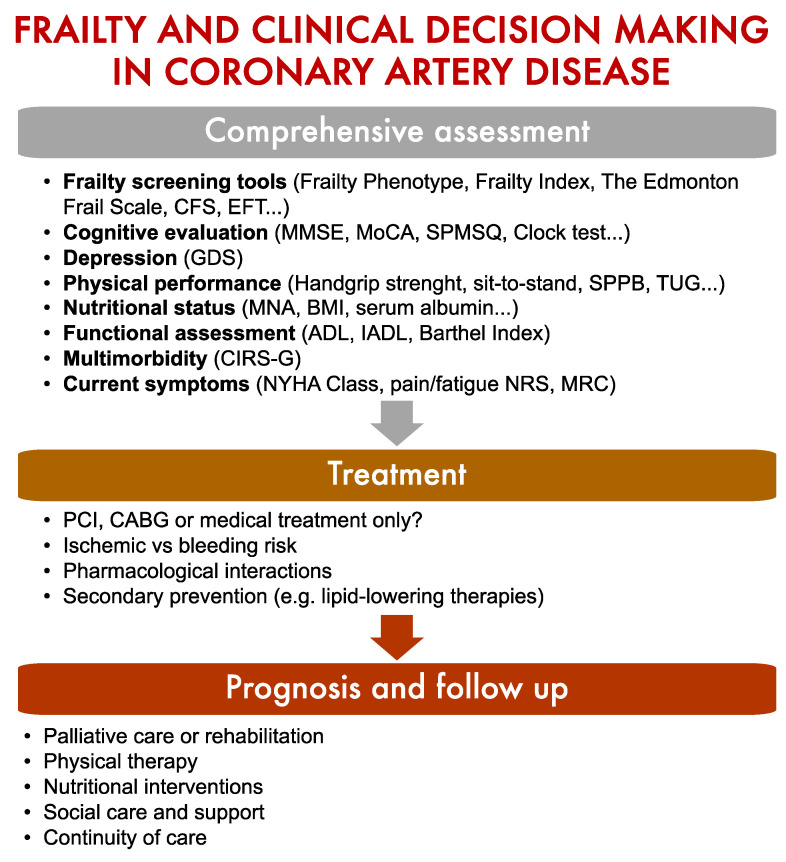
Screening for frailty is essential to guide clinical decision-making in older adults with coronary artery disease. Abbreviations: BMI: Body Mass Index; CABG: Coronary Artery Bypass Grafting; CFS: Clinical Frailty Scale; CIRS-G: Cumulative Illness Rating Scale-Geriatric; EFT: Essential Frailty Toolset; GDS: Geriatric Depression Scale; MMSE: Mini Mental State Examination; MNA: Mini Nutritional Assessment; MoCA: Montreal Cognitive Assessment; MRC: Medical Research Council Dyspnoea Scale; NRS: Numerical Rating Scale; NYHA: New York Heart Association; PCI: Percutaneous Coronary Intervention; SPMSQ: Short Portable Mental State Questionnaire; SPPB: Short Physical Performance Battery; TUG: Timed Up and Go Test.

Frailty is a complex age-related condition frequently experienced by older adults with CVD, and is associated with a high risk of negative outcomes [5]. Although there is no univocal operational definition of the condition, frailty can be conceived as a syndrome with multiple causes and contributors that is characterized by diminished strength, endurance, and reduced functional reserve that increases an individual’s vulnerability to develop dependency and/or die [6]. Thus, the assessment of frailty is expected to provide relevant information to guide therapeutic decision-making and follow-up (Figure 2).

Frailty is strongly and independently associated with a greater risk of negative short-term clinical outcomes in older patients with non-ST-elevation (NSTEMI)-ACS [7]. According to the most recent European guidelines, the clinical decision-making in this setting relies primarily on cardiovascular risk [8]. However, increasing evidence suggests that the performance of an invasive approach for NSTEMI-ACS in older adults may better predicted by the geriatric evaluation of frailty and comorbidities [7]. A recent meta-analysis revealed that frailty was extremely common in patients with ischemic heart disease, suggesting that it can become a useful tool to better characterize this condition [9].

Although there are different ways to operationalize frailty, two main models have been proposed. The frailty phenotype devised by Linda Fried [10] is based on the presence of five signs/symptoms (i.e., unintentional weight loss, fatigue, weakness, slowness, and reduced physical activity). On the other hand, the Frailty Index (FI), elaborated by Kenneth Rockoowd [11], is built on the conceptualization of frailty as the accumulation of health deficits. A simple and quick tool to evaluate frailty is the Clinical Frailty Scale (CFS) based on pictograms accompanied by a short description [12]. The EFT (Essential Frailty Toolset) is a simple validated tool to detect frailty and the risk of mortality or disability after a transcatheter aortic valve replacement or surgical aortic valve replacement procedure, taking into account four items (lower-extremity weakness, cognitive impairment, anemia, and hypoalbuminemia) [13].

Multidimensional approaches may also be used for frailty assessment. For example, the Edmonton Frail Scale is a relatively simple and validated tool that explores cognition, prior hospital admissions, self-rated health, functional independence, social support, medication use, nutritional status, mood, continence, and physical performance [14]. The multidimensional assessment can be further deepened through the evaluation of the impact of comorbidities (e.g., Cumulative Illness Rating Scale for Geriatric Patients [15]), disability and functional status (e.g., activities of daily living and instrumental activities of daily living scores [16], Barthel Index [17]), cognitive status (e.g., Mini Mental State Examination [18], Montreal Cognitive Assessment [19], Short Portable Mental Status Questionnaire [20]), as well as sarcopenia and physical performance. The latter are relevant metrics to assess overall health and predict outcomes and, as such, should be part of a multidimensional evaluation [21,22]. Sarcopenia—the loss of skeletal muscle mass, strength and function [23]—is a powerful predictor of CVD progression, falls, reduced quality of life, and death in patients with CVD [24,25,26,27,28]. The presence of sarcopenia can be explored by the handgrip strength test and estimation of lean body mass through imaging or anthropometric measures. A decrease in physical performance as assessed using the Short Physical Performance Battery (SPPB), timed-up-and-go test, and self-reported mobility limitations is used as a criterion of severity [23].

Nutritional status should be investigated since malnutrition is associated with higher mortality and in-hospital complications in patients with CAD [29,30]. The Global Leadership Initiative on Malnutrition (GLIM) recommends a two-step approach that involves appraisal of the likelihood of malnutrition and its subsequent identification [31]. The Mini Nutritional Assessment (MNA) can be used to screen malnutrition [32]. However, the body mass index (BMI) may also be a useful proxy to assess nutritional status in older patients [33]. Finally, hypoalbuminemia (<30 g/L) may identify severe malnutrition [34].

Depression is frequent in older patients and significantly increases the risk of CAD [35]. A quick assessment of depression may be achieved by using the Geriatric Depression Scale (GDS) [36].

In conclusion, frailty is a major predictor of adverse outcomes in older patients with CAD and should always be assessed in this population. As recommended by Boreskie et al. [37], frailty assessment should be tailored to the intensity of care in the clinical setting and available resources. Both cardiologists and geriatricians should be familiar with the concept of frailty and its implications and should conduct a proper evaluation when necessary.

## 3. Optimal Strategy during the Acute Phase of Coronary Syndromes: Percutaneous Coronary Intervention, Coronary Artery Bypass Graft Surgery, or Medical Treatment?

**Figure 3:**
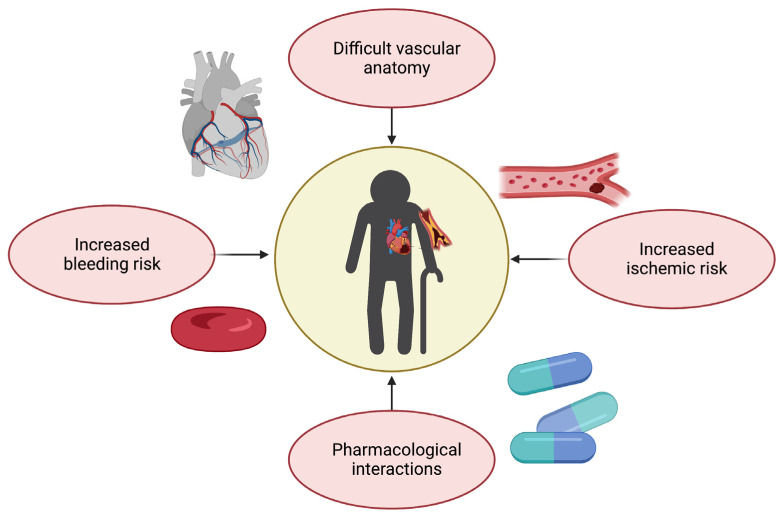
Challenges in therapeutic management of coronary artery disease in older adults (created with Biorender.com, accessed on 30 June 2023).

As previously discussed, the management of CAD in older adults requires a multidimensional clinical approach that goes beyond pre-defined therapeutic and nosographic algorithms (Figure 3). For instance, older adults are at high risk of both ischemic and bleeding events [38]. Multivessel disease is also frequent in old age [39]. Furthermore, advanced age is associated with adverse outcomes across the whole spectrum of ACSs, partly because of their frequent atypical presentation, which may delay their recognition and treatment [39].

Guidelines recommend the use of predictive tools such as the Thrombolysis in Myocardial Infarction (TIMI), Global Registry of Acute Coronary Events (GRACE) or GRACE 2.0 for risk assessment and management [8]. The TIMI score was designed to assess the risk of unfavorable outcomes in patients with ACS; however, its reliability in geriatric populations has been somewhat restricted [40]. The GRACE score has been validated in older adults; nevertheless, its accuracy in this subgroup might be reduced due to competing factors [41]. Interestingly, a study on 198 patients with type 1 myocardial infarction conducted by Anand et al. found that while the GRACE score alone overestimated mortality risk, a simple frailty screening tool such as the CFS was an independent predictor of mortality and significantly enhanced the GRACE 12-month mortality estimate [42]. GRACE 2.0 demonstrated better discrimination than the prior version and functioned equally well in acute and long-term circumstances [43]. According to Hung et al., GRACE 2.0 demonstrated high accuracy for prognostic stratification of patients with type 1 myocardial infarction and intermediate accuracy for those with type 2 myocardial infarction, who are often older and have more comorbidities [44].

Despite the underrepresentation of older patients in landmark clinical trials on ACS and the consequent lack of specific pharmacological and invasive treatment recommendations, the application of existing guidelines reduces mortality after hospital admission in this specific patient subgroup [45]. This may be due to an increasing application of invasive approaches to ACS in aged individuals, which showed a better benefit–risk ratio compared with conservative treatments in the setting of both ST elevation (STEMI)- and NSTEMI-ACS [46,47]. Indeed, current European guidelines recommend applying the same invasive approaches in older adults as in younger patients. In the setting of STEMI-ACS, European guidelines recommend coronary angiography with primary percutaneous coronary intervention (pPCI) in patients of all ages within two hours of symptom onset. Within this timeframe, a pPCI strategy is recommended over fibrinolysis—otherwise, patients may receive fibrinolysis—and those ineligible for any reperfusion strategy should be treated medically with dual antiplatelet therapy [8]. In an emergency setting, coronary artery bypass graft (CABG) surgery is limited to patients with ongoing ischemia with unsuitable anatomy for a percutaneous approach [8]. In the case of NSTEMI-ACS, European guidelines recommend performing coronary angiography and subsequent revascularization, if indicated, in patients at intermediate or higher risk of adverse outcomes, regardless of age [8]. Surgery is considered a more suitable option in patients with diabetes mellitus or complex multivessel disease and may become the only approach in case of coronary anatomy not amenable to PCI, unsuccessful PCI, or when surgical treatment of mechanical complications or concomitant valve disease is mandatory [8,48,49]. However, although these indications may be valid in more stable and more robust patients, in the majority of cases, surgical treatment of ACS has a very high risk both in the short and the long term [50], especially in aged individuals [51]. For this reason, surgical treatment is now very uncommon in clinical practice, especially in older patients.

Sedation plays an important role in ensuring the comfort and safety of PCI patients. However, administering sedation to older persons necessitates careful evaluation of possible hazards due to increased sensitivity to sedatives as a result of multimorbidity, as well as age-related changes in metabolism and clearance, which may raise the risk of oversedation, adverse reactions and delirium. Delirium is significantly associated with in-hospital mortality and an increased risk of postprocedural complications [52]. A recent expert panel of the American College of Cardiologists advised against using sedatives with a prolonged half-life, such as diphenhydramine and long-acting benzodiazepines [53]. Studies identify dexmedetomidine as a potential alternative for older patients because it demonstrates non-inferiority for light-to-moderate sedation compared to midazolam and propofol and decreases the occurrence of delirium, despite the development of hypertension, bradycardia, and tachycardia [54]. Although sedation-free protocols could reduce days without mechanical ventilation in critically ill patients, it was associated with higher risk of delirium [55]. To date, however, sufficient evidence on the optimal sedation strategy for older patients is lacking [56]. Dosing the serum [57] or cerebrospinal fluid biomarkers [58] may provide a new tool to guide decision-making for preventing delirium in the cardiac intensive care unit in the near future, although it has not yet proven to be sufficiently specific for this goal. For now, in older individuals, the appropriate sedation technique to ensure the greatest balance between patient comfort and hemodynamics may differ from patient to patient, depending on comorbidities, frailty, and general health state.

## 4. Medical Treatment

The optimization and dosage of all drugs is of utmost importance in older frail patients, with particular attention to antithrombotic agents, owing to the risk of side effects and drug interaction [59]. Age-related changes in pharmacokinetics and pharmacodynamics—potentially due to changes in the distribution of fat mass and lean mass, multimorbidity, and polypharmacy—are associated with an increased risk of drug toxicity and side effects in older patients [60]. Sarcopenia, for instance, may cause underestimation of glomerular filtration rate calculated using serum creatinine, leading to inappropriate direct oral anticoagulant (DOAC) dosing and increased risk of bleeding [61]. Based on these observations, while aspirin remains the cornerstone for secondary CVD prevention [62], American guidelines do not endorse its use on a routine basis for primary prevention among adults over 70 years [63]. In particular, when prescribing dual antiplatelet treatment after ACS or PCI, it is pivotal to tailor its duration in order to maximize ischemic protection while limiting bleeding risk, even though this may be challenging due to an overlap between ischemic and bleeding risk in frail patients (Figure 3) [60]. However, in both the PRECISE-DAPT and the DAPT scores, as well as according to the Academic Research Consortium for High Bleeding Risk (ARC-HBR) consensus, age is an important parameter that tips the scale towards short dual antiplatelet treatment regimens [44,64,65]. Among P2Y12 receptor inhibitors, prasugrel is generally not recommended in patients over 75 since the TRITON-TIMI 38 trial reported excess bleeding risk, resulting in a neutral net clinical benefit in older patients [66]. Conversely, the use of ticagrelor is not restricted to aged patients after ACS, based on the results of the PLATO trial [67]. Similarly, no restrictions based on age for long-term ticagrelor use on top of aspirin are recommended in patients with previous spontaneous myocardial infarction deemed to be at high ischemic risk, based on the PEGASUS-TIMI 54 trial [68]. Eventually, the POPular AGE trial identified clopidogrel as a favorable alternative to ticagrelor in older patients with high bleeding risk, due to fewer bleeding events and non-inferiority in the combined endpoint of all-cause death, myocardial infarction, stroke, and bleeding [69]. The trade-off between bleeding and ischemic risk becomes more challenging if we consider that 20–30% of older patients with atrial fibrillation (AF) need PCI and stenting for concomitant CAD. Indeed, a triple antithrombotic treatment (aspirin, P2Y12, and anticoagulant) has been associated with an almost four-fold higher risk of bleeding than oral anticoagulation (OAC) monotherapy [70,71]. Several studies have compared dual (i.e., single antiplatelet therapy with a P2Y12 inhibitor plus OAC) with triple antithrombotic therapy with regard to bleeding drawbacks. Recently, an important meta-analysis of pooled data from three major randomized trials reported that dual antithrombotic treatments including DOACs and a P2Y12 inhibitor without aspirin were associated with significantly lower bleeding than vitamin K antagonist (VKA)-based triple antithrombotic therapy in AF patients undergoing PCI [55,72,73,74]. Hence, after an initial short period (up to one week in NSTE-ACS and stable CAD) of triple antithrombotic therapy with DOAC and dual antiplatelet treatment, in most old and frail patients with concomitant AF, dual antiplatelet therapy is recommended as the default strategy using a DOAC at the recommended dose for stroke prevention and a single oral antiplatelet agent (preferably clopidogrel) [8,75]. Nevertheless, it should be considered that frailty is an independent predictor of bleeding, and treatment should be carefully tailored to each patient’s risk–benefit balance [55,76]. The most recent evidence supports de-escalation strategies, which can be achieved in several ways [60]. First, one possible strategy could be shortening DAPT followed by aspirin, clopidogrel or ticagrelor monotherapy. Other options include guided strategies implementing platelet function testing or genetic testing [77] as well as unguided de-escalation [78,79].

Certain antidepressants interact with antithrombotic treatment, leading to an increased risk of bleeding by blocking platelet uptake of serotonin [80]. Therefore, the use of nonselective serotonin re-uptake inhibitors may be proposed, or a proton pump inhibitor may be prescribed in high-risk bleeding patients treated with selective serotonin re-uptake inhibitors.

Additional goals of medical therapy for CAD are to relieve symptoms, reduce cardiac workload, and prevent complications. For these purposes, recommended medications include nitrates, beta blockers, angiotensin-converting enzyme (ACE) inhibitors or angiotensin II receptor blockers (ARBs), and statins [8]. However, their administration in older patients requires careful consideration due to several concerns.

Nitrates effectively relieve angina symptoms and improve coronary blood flow in ACS patients. They can help reduce the ischemic burden on the heart and provide symptomatic relief, thereby improving quality of life [81]. However, nitrates can cause a drop in blood pressure, leading to hypotension [82]. Older patients may be more susceptible to this side effect due to age-related changes in blood vessel elasticity and autonomic regulation, interactions with concomitant medications, as well as pre-existing conditions [83]. Therefore, a cautious monitoring of blood pressure is necessary when initiating nitrates in older patients, especially in those with pre-existing hypotension or orthostatic hypotension [84].

Together with nitrates, it is generally recommended to initiate beta blockers early in the management of ACS, ideally within the first 24 h, unless there are contraindications or specific patient factors that warrant delay [8]. Benefits of beta blockers include reducing myocardial oxygen demand, decreasing heart rate and blood pressure, preventing arrhythmias, and improving long-term outcomes [85,86]. However, they may be contraindicated or require cautious use in certain situations. For example, in patients with severe bronchospastic disease, nonselective beta blockers should be avoided or used with extreme caution due to the potential for exacerbating bronchospasm. The choice of specific beta blocker should be guided by the patient’s comorbidities and tolerability [87]. For example, if a patient has a history of heart failure, a beta blocker with additional alpha-blocking properties like carvedilol may be preferred [88].

ACE inhibitors and ARBs reduce mortality, prevent heart failure, and improve outcomes in ACS patients [89,90]. A decline in renal function in older patients may affect the metabolism and elimination of both ACE inhibitors and ARBs. Moreover, aged individuals may have an increased risk of developing hyperkalemia due to an age-related decline in renal function and comorbidities such as diabetes [91]. Dose adjustments and close monitoring of renal function and electrolyte levels are important, especially in patients with pre-existing renal impairment or those taking other medications that can increase potassium levels [92].

Statins are the mainstay of lipid-lowering therapy and have been extensively studied in ACS patients [93]. However, their use in older patients, especially over the age of 75, in primary prevention is a matter of ongoing debate. As highlighted by a systematic review and meta-analysis by Aeschbacher-Germann and colleagues [94], participants enrolled in most clinical trials on lipid-lowering therapies are not representative of the general population. Statin therapy should be guided by the patient’s risk profile, baseline low-density lipoprotein (LDL) cholesterol level, tolerability, and predicted long-term benefits. Statins are metabolized by the cytochrome P450 (CYP450) enzyme system (except for pravastatin, rosuvastatin, and pitavastatin) [95]. Competing factors such as interactions with medications that inhibit or induce the CYP450 system or reduced renal function, may increase circulating levels or decrease the effectiveness of statins [96,97,98,99,100]. Although the efficacy of statins may be questionable for primary prevention in adults older than 75 [101,102], their use at an appropriate (not suboptimal) dosage is effective in secondary prevention, and that is clearly presented in the available guidelines [103,104,105]. However, a recent meta-analysis of 10 observational studies with 815,667 primary prevention patients showed that statin therapy was associated with a significantly lower risk of all-cause mortality (hazard radio (HR) 0.86, 95% confidence interval (CI) 0.79–0.93), CVD death (HR 0.80, 95% CI 0.78–0.81), and stroke (HR 0.85, 95% CI 0.76–0.94) and non-significantly associated with risk of myocardial infarction (HR 0.74, 95% CI 0.53–1.02). The beneficial association of statins with the risk of all-cause mortality remained significant even at older ages (>75 years; HR 0.88, 95% CI 0.81–0.96) and in both men (HR 0.75, 95% CI 0.74–0.76) and women (HR 0.85, 95% CI 0.72–0.99) [106]. The STOPPFrail (Screening Tool of Older Persons Prescriptions in Frail adults with Limited Life Expectancy) consensus highlights that lipid-lowering therapies need a long time to provide benefits. For this reason, potential risks may outweigh benefits if administrated for a short period in older patients with limited life expectancy [107]. Further trials using composite endpoints may help better understand benefits of statins in older populations, while bempedoic acid could be a therapeutic option to overcome safety issues related to statin intolerance [108]. It is worth remembering that older age, per se, is a risk factor for statin intolerance (by even 31–33%) [109]. For this reason, a stepwise lipid lowering approach is indicated, starting with moderate-intensity statin therapy, or, in case of any adverse events, considering lipid-lowering combination therapy with an ezetimibe, bempedoic acid and PCSK9 targeted therapy approach, for which there is strong evidence of the safety and efficacy, including in aged populations [110].

In conclusion, when considering medical therapy in older adults with CAD, several important factors should be taken into account. These include the patient’s overall health status, comorbidities, functional limitations, and goals of care. Older adults may have age-related changes in drug metabolism, increased susceptibility to medication side effects, and a higher burden of polypharmacy. Therefore, a personalized approach to medical therapy is crucial, involving careful selection and titration of medications, regular monitoring for adverse effects, and frequent follow-up visits. To mitigate the risk of drug interactions in older patients with CAD, comprehensive medication reviews, including an assessment of the patient’s complete medication list, should be conducted regularly. A close monitoring for potential interactions, regular communication among healthcare providers, and patient education about their medications are essential to optimize treatment outcomes while minimizing the risks associated with drug interactions [111]. Collaboration between healthcare professionals, including cardiologists, geriatricians, and primary care physicians, is essential to ensure the optimal management of CAD in older adults, promoting both cardiovascular health and overall wellbeing [111].

## 5. Follow-Up of Older Patients and Collaborative Approach

In a recent scientific statement concerning the management of ACS in the older population, the American Heart Association (AHA) acknowledges the possibility of suboptimal care transitions as a contributing factor to the decline in independence [112]. Consequently, individuals experiencing an ACS need a comprehensive and vigilant post-treatment regimen encompassing the monitoring of symptom severity, functional capacity, and overall health-related quality of life [112,113]. It is important to point out that recently hospitalized patients are not only recovering from their acute illness but also experiencing a transient period of generalized risk for a wide range of adverse health events [114]. Following discharge, as well as during the subsequent 30-day period, the body’s ability to effectively prevent or mitigate health threats is compromised due to a depletion of physiological reserves [114]. This phenomenon, commonly known as the “post-hospital syndrome”, predominantly affects older and complex patients [114]. Up to now, follow-up modalities for these patients are not well-defined and standard protocols have not yet been established. In an observational study conducted by Lettieri et al. [115], an attempt was made to evaluate several aspects, including the frequency and patterns of cardiology visits, echocardiographic examinations, and stress tests following PCI in real-world clinical settings. The study also sought to assess the effects of a multidisciplinary protocol for long-term post-PCI follow-up, which involved collaboration with general practitioners, with a particular focus on its impact on the appropriateness of care and potential reduction in healthcare costs [115]. A total of 780 patients, with a mean age of 67 years, were eligible for inclusion in the study. Findings revealed a considerable variation in follow-up strategies, leading to a significant level of inappropriateness across various outpatient services and resulting in higher healthcare costs [115]. Upon analyzing a subgroup of 305 patients who did not undergo any provocative tests within a two-year period following PCI, it was observed that they were significantly older compared with those who underwent functional tests (mean age of 71 years vs. 64 years, respectively) [115]. Moreover, this subgroup exhibited a higher prevalence of more severe comorbidities, making them unsuitable candidates for functional tests [115]. Consequently, in multimorbid and complex individuals, it is pivotal to tailor follow-up of ischemic heart disease by employing the most suitable provocative test. Regarding the medical approach, the type and extent of follow-up should be contingent upon several factors, including the patient’s disease stage, anticipated lifespan, personal preferences, comorbidities, and regional healthcare organization [116]. Particular attention should be paid to potential challenges that older patients may encounter during the post-discharge phase, such as living alone, lack of a social support network, and cognitive decline, which potentially impact therapeutic adherence [112,117]. For this reason, following discharge for an ACS, several key aspects warrant optimization. These include medication management, including simplification of therapeutic regimens and deprescription whenever feasible [118]. Lifestyle modifications and engagement in cardiac rehabilitation are also essential, along with the effective management of comorbidities [112]. Adequate psychosocial support, consideration of socioeconomic factors, and proactive prevention of adverse events such as functional decline, rapid hospital readmission, and mortality are crucial [119]. Equally important is the provision of patient and family education to promote self-care practices [116,119]. Among the challenges encountered, one of significant importance lies in achieving physical reactivation or resuming training, with particular emphasis on including regular physical activity within the daily routine [116,120]. Coping with all these issues may be difficult, especially for older patients and their caregivers, who must usually manage other concomitant diseases, with many different specialists at the same time. Considering all these reasons, directing older patients towards cardiac rehabilitation (CR), whether in a residential or ambulatory setting, could prove to be a favorable decision [121,122]. Such an approach can effectively streamline the transition from hospital to community care while ensuring a personalized and comprehensive treatment plan [121,123]. This is made possible by a multidisciplinary team capable of addressing the patient’s needs from various perspectives [121,122,123]. The primary goal of CR interventions is to prevent the onset or progression of frailty or disability and preserve the remaining functional capacity [121]. Throughout the program, including admission, discharge, and follow-up visits, patients should undergo a comprehensive assessment encompassing cardiological, clinical, functional, emotional, cognitive, and social domains [121,123]. This multidimensional evaluation allows for a comprehensive appraisal of the patient’s condition and aids in tailoring the rehabilitation program accordingly [121,122]. A systematic review conducted by Khan et al. [124] revealed a high prevalence of depression among patients following ACS, emphasizing a significant undertreatment of this condition. This attitude has been linked to increased morbidity and mortality, primarily attributable to reduced adherence to guideline-directed therapies, self-care practices, and clinic visits [112,124]. Consequently, it is imperative to encourage and provide patients with the opportunity to engage in CR programs. Such programs can effectively improve their functional performance and enhance exercise tolerance. Very old and frail patients may obtain greater benefits from participating in CR programs: initiating these programs early after an acute event has the potential to prevent the onset of post-hospital syndrome [9,125]. In case of home discharge, additional challenges may be faced, particularly due to a frequently inadequate communication between hospital cardiologists and other healthcare professionals [116]. It is common for patients to be discharged with a written letter that, although explained to patients and their families, is often not thoroughly discussed with the actual caregivers; this situation poses a risk of crucial information regarding behavioral and therapeutic guidelines being lost [112]. To ensure optimal care coordination, the multidisciplinary team should include various healthcare providers such as cardiologists, surgeons (when applicable), primary care clinicians, geriatricians, nurses, and social workers, as well as the patient’s family or significant others [112]. A ready access to professionals such as pharmacists, dieticians, psychologists, occupational therapists, and case managers, as needed, further contributes to comprehensive care management [112]. The cognitive status of the patient is another important element to consider. Cognitive impairment (CI) is prevalent among patients with ACS during both the early recovery phase and the long term, although it is currently inadequately characterized [126]. CI often goes undetected and has the potential to progress to dementia [126]. The impact of CI on a patient’s ability to comprehend health education and adopt behavioral changes following ACS is not well understood but may hold significant importance [126]. In the acute setting, individuals with mild CI (MCI) or pre-existing dementia may experience further deterioration in cognitive function beyond their baseline level due to the stress of the acute event, an unfamiliar environment, or side effects of medications [126]. In a comprehensive multilevel study, data from two databases were included, namely later-stage elderly healthcare insurance and long-term care insurance claims, spanning the period from 2013 to 2019 [127]. Among a total of 214,963 individuals diagnosed with dementia, 13,593 experienced an acute myocardial infarction (AMI) [127]. Findings from this study suggest that combining long-term care with invasive procedures may offer a promising management strategy for AMI among patients with dementia, potentially leading to a reduction in mortality risk [127]. Therefore, clinicians should gain awareness regarding the varying effects of CI across different cognitive domains and adapt their management strategies accordingly. It is crucial to implement regular screening tests that assess both global and higher-order cognitive functions in older patients with heart failure [128]. By doing so, optimal support for self-care can be provided, recognizing the specific cognitive areas that require attention and intervention [128]. Eventually, it is worthwhile considering the setting of patients who are approaching the end-of-life phase. For these patients, the AHA recommends focusing on outcomes such as avoiding re-hospitalizations and relieving pain and discomfort [112]. A multidisciplinary discussion may aid in determining a treatment’s futility [112]. Before invasive procedures, do-not-resuscitate orders should be carefully discussed with the patient, family, or power of attorney [112].

## 6. Gaps in Evidence

No dedicated randomized controlled trials (RCTs) have yet assessed the effectiveness of a management strategy based on a risk-prediction model (such as the PRECISE-DAPT score or ARC-HBR criteria) for determining the duration of dual antiplatelet therapy following PCI for NSTEMI-ACS [8]. However, although older patients are generally underrepresented in clinical trials, the number of studies involving aged individuals has grown in recent years [112].

Patients over 80 years were involved in the After Eighty [129] and SENIOR-NSTEMI [130] trials, which demonstrated benefits of invasive treatments versus conservative approaches. The Italian Elderly ACS trial [131], the MOSCA trial [132] and a randomized clinical trial by Hirlekar and colleagues [133] involved patients 75, 70, and 80 years or older, respectively, and did not find long-term advantages of invasive approaches for NSTEMI-ACS. The RINCAL randomized clinical trial reported a non-superiority of invasive treatments versus optimized medical therapy alone in ultra-octogenarians with NSTEMI [134], and unpublished results from the MOSCA-Frail RCT did not observe benefits of invasive approaches in aged patients with NSTEMI [112,135].

In these studies, frailty assessment was performed only in the MOSCA-Frail trial and in the study conducted by Hirlekar and colleagues [133,135]. While the first used CFS [136], the latter evaluated frailty through the Canadian Study of Health and Aging Clinical Frailty Scale. However, both studies reported a considerably low proportion of frail individuals and did not stratify results according to frailty status [133].

Multicenter RCTs with an adequate representation of older patients are needed to assess the safety and effectiveness of different treatment strategies in this population [8]. The lack of a widely used frailty assessment tool is a significant limitation in trials examining the therapeutic management of ACS in older adults [2]. Achieving better collaboration and coordination among the various clinicians involved in the care of older ACS patients remains a challenging task that is of crucial importance, as pointed out previously. To that end, it may be worthwhile developing and testing dedicated multidisciplinary management programs for older ACS patients [112].

## 7. Future Perspectives and Conclusions

Research is needed to accurately determine the prevalence of CI in ACS patients and create suitable standardized measures and thresholds for future practice in the prediction of recurrences [126].

A systematic review and meta-analysis by Khoong et al. [136] assessed the effectiveness of mobile health strategies for self-management of hypertension in vulnerable populations experiencing outcome disparities (older, minority, and limited educational attainment patients). The authors pointed out that blood pressure control is often worse in those populations that are underrepresented in digital health studies [136]. Furthermore, many trials have shown that post-hospital telecardiology improves outcomes and reduces re-admissions or outpatient contacts in those with heart failure, arrhythmias, or implantable devices. Again, however, the literature on geriatric patients is sparse [137]. Telecardiology (e.g., home electrogram) may prove useful in avoiding delays to treatment and a wrong diagnosis in the case of STEMI. Benefits may even increase for older patients, who often present with symptoms other than chest pain [138,139]. This evidence reinforces the need to specifically include diverse populations in high-quality clinical trials on digital health. Although promising and suggestive, the implementation of telemedicine in geriatric patients may face numerous obstacles related to issues of social frailty, digital illiteracy, or poor adherence [140,141]. Instead, comprehensive geriatric assessment and multidisciplinary care involving cardiologists, geriatricians, nurses, physical and occupational therapists, as well as caregiver education, could bring several benefits in emerging symptom management, prevention of inappropriate or avoidable hospitalizations, and CR [142,143,144].

The primary frontier in the management of the older patient with ACS is the transition from a disease-centered model to person-centered approaches. A cost-effectiveness study conducted concurrently with a randomized clinical trial in adults over the age of 65 revealed favorable health and cost implications in 90% of cases [145]. Patient-centered care entails making informed choices together with the patient and all stakeholders and establishing goals and expectations to define clinical treatment. In research, all of this involves selecting appropriate outcomes to measure the true impact of an intervention [146].

In the future, rather than considering frailty as a criterion for treatment exclusion, improving our knowledge of this condition should lead to the development of tailored management aimed at achieving the best possible care in older persons with ACS.

## Data Availability

Not applicable.
